# Science with Seymour Levine: on glucocorticoid action from birth to senescence

**DOI:** 10.1016/j.ynstr.2026.100801

**Published:** 2026-03-17

**Authors:** E.R. de Kloet

**Affiliations:** Leiden University Medical Center, Department of Clinical Medicine, Division of Endocrinology, Leiden, the Netherlands

**Keywords:** Development, Stress, Adaptation, Brain, Glucocorticoid, Cortisol, Glucocorticoid receptors

## Abstract

This tribute to the late Seymour Levine, marking his 100th birth year, highlights the following discoveries in rodents that we had the privilege of sharing with him. (i) Cessation of glucocorticoid receptor (GR) expression in the suprachiasmatic nucleus (SCN) upon innervation by the optic nerve around postnatal day 12 may explain phase dissociation of the master pacemaker from glucocorticoid-driven cellular clocks under, e.g., conditions of chronic stress. (ii) Maternal care protects the infant from noxious influences, while preparing it for stress coping in future life, for better and worse, i.e., the predictive adaptive response. (iii) The feeding and tactile components of maternal care allow to distinguish the activation of central from adrenal glucocorticoid-dependent components of the pup's stress response system. This knowledge helps understand how the amygdala-mediated fear response is primed. (iv) Maternal separation for 24 h at postnatal day 3 drives cognitive aging to either senility or excellence at the expense of partially impaired cognitive performance that is characteristic of a normal aging trajectory. The findings inform how glucocorticoid action during early-life experience can amplify individual variation in stress coping and adaptation from birth to senescence.

## 1 Introduction

Seymour Levine, also known as Gig to insiders, was a frequent guest of David de Wied, who directed the Rudolf Magnus Institute at Utrecht University in the Netherlands from 1963 to 1990, and investigated the behavioral pharmacology of neuropeptides related to ACTH, vasopressin, and oxytocin. Although I occasionally met Gig in the Institute, it was not until 1986 at the 17th Congress of the International Society of Psychoneuroendocrinology (ISPNE) in Bergen, Norway, organized by Holger Ursin and Robert Murison, that a close collaboration began to develop.

Around that time, we had just discovered that in the rodent, the endogenous glucocorticoids corticosterone and cortisol (collectively called CORT) bind not only to the classical glucocorticoid receptor (GR), but also with much higher affinity to the CORT-preferring mineralocorticoid receptor (MR) (Reul and de Kloet, 1985) (BOX 1). Seymour Levine was the Symposium's chairman, where I presented the data, and he immediately recognized the significance of the complementary MR/GR-mediated actions in the brain. He convinced one of his outstanding students, Patricia Rosenfeld, to pursue her PhD in Utrecht, starting in the fall of 1986, and frequently monitored progress in person while participating in the experiments. It was a marvelous adventure exploring with Gig the ‘*laboratory of nature*’ as he used to describe experimentation in the developing brain.

BOX 1 **Complementary MR- and GR-mediated actions**

(i) The corticosterone-preferring MR type is expressed at very high abundance in the lateral septum, hippocampal pyramidal and dentate gyrus neurons, and at lower levels in neurons of the amygdala and layer 2 of the neocortex (Ahima et al., 1991; Arriza et al., 1988; Reul and de Kloet, 1985). This MR is the high-affinity corticosterone binding site discovered by Bruce McEwen (McEwen et al., 1968). The distribution of the lower-affinity GR is uneven but ubiquitous in the brain (Ahima and Harlan, 1990; Cintra et al., 1994; van Eekelen et al., 1987). In the mid-1980s, MR and GR were cloned by Ron Evans (Arriza et al., 1987; Evans and Arriza, 1989; Hollenberg et al., 1985).
(ii) MR, which is always substantially occupied by corticosterone (CORT) maintains basal HPA axis activity. MR is, along with GR, involved in the ***proactive*** control of daily activities, sleep-related events, and synchronization of the circadian cellular clock (Oster, 2020; Oster et al., 2016).
(iii) MR is ***proactive*** in the onset of the stress response and defense systems, which are essential for information retrieval, appraisal processes, and selection of an appropriate coping style. GR operates in a ***reactive*** mode, responsible for turning off the stress response, promoting energy metabolism, controlling defense reactions to prevent them from overshooting, enhancing emotional and motivational arousal, and storing experience and energy substrates for future use, to support adaptation (de Kloet, 2014; De Kloet et al., 2005; de Kloet et al., 1998; de Kloet and Joëls, 2024).
(iv) CORT-preferring MR (see Box 4) and GR act together to regulate cellular and system responses with mechanisms extending from slow genomic responses to fast non-genomic actions (Di et al., 2003; Herman and Tasker, 2016; Joëls et al., 2012, 2024; Karst et al., 2005, 2010). The balance between the complementary actions of CORT, mediated by MR and GR, is crucial for adaptation and health (de Kloet, 2014; de Kloet et al., 1998, 2005, 2018; de Kloet and Joëls, 2024; Hartmann et al., 2021; Daskalakis et al., 2022).

Alt-text: BOX 1

An AI inquiry on early life programming answers within a split second: “*The concept of early handling in rats, where handling infant rats reduces their adult fear responses, was significantly developed by researchers like Seymour Levine and his colleagues in the mid-1950s*”. In honor of Gig's 100th birth year, I will highlight several lines of our past research -mostly with male infants-that were initiated while he was available for advice and collaboration. This research culminated in a better understanding of how pups' early-life experience, maternal care, and CORT contribute to the synchronization of circadian organization and later-life resilience. For reviews on Seymour Levine's legacy, see, among others (Cirulli and Suchecki, 2010; Coe et al., 2008; Stern et al., 2010; Suchecki, 2018).

## 2 Circadian rhythmicity

**Rat suprachiasmatic nucleus (SCN) neurons express GR during the rodent perinatal period until the optic nerve input is established around PND 12; thereafter, SCN-GR expression fades** (Van Eekelen et al., 1987a; Rosenfeld et al., 1988, 1993b)**. This is the outcome of our initial collaborative studies with Gig in the late 1980s. The glucocorticoid-driven cellular clocks are proposed to dissociate from the GR-devoid SCN master pacemaker during jet lag, shift work, and chronic stress. Such CORT-driven desynchronization of cellular clocks may compromise resilience** (Balsalobre et al., 2000; Conway-Campbell et al., 2010; Sarabdjitsingh et al., 2010; Lehmann et al., 2023)**.**

### 2.1 Organization of circadian clocks

The circadian rhythm is characterized by a surge of CORT that mobilizes energy towards the active period, which occurs at dusk in rodents and at dawn in humans. The SCN drives this surge in CORT, as its ablation in rodents eliminates the circadian rhythm (Buijs and Kalsbeek, 2001; Moore and Eichler, 1972). Subsequent studies showed that the SCN controls the CRH synthesis in the paraventricular nucleus (PVN) via an inhibitory or excitatory input, including also vasopressin and vasoactive intestinal peptide, irrespective of a nocturnal or diurnal species, a mechanism that was further elaborated using optogenetics (Kalsbeek and Buijs, 2002; Ono et al., 2021; Ramirez-Plascencia et al., 2025). The amplitude of the circadian CORT rhythm is regulated through an SCN-derived sympathetic sensitization of the adrenal gland to ACTH (Engeland and Yoder, 2012).

The mechanism underlying the circadian rhythm is based on light-induced signaling from the retina to the SCN, which induces the synthesis of the Circadian Locomotor Output Cycles Kaput (CLOCK) and Brain and Muscle ARNT-Like 1 (BMAL1) proteins in both nocturnal (rodents) and diurnal species (humans). These two proteins form a heterodimer that increases the synthesis of the PER (period) and CRY (cryptochrome) transcription factors, in a negative feedback loop with the original CLOCK: BMAL1 genes over a 24-h period (Oster, 2020). Interestingly, PER1 expression in the hippocampus rapidly responds to hourly CORT variations, with a short half-life, characteristic of ultradian rhythmicity. This implies that disruptions of ultradian and circadian CORT rhythmicity would have immediate consequences for CORT responsivity in hippocampus-dependent learning and memory function (Conway-Campbell et al., 2010; Sarabdjitsingh et al., 2010).

The 24-h clock mechanism is present in every cell of the brain and body, but differs from that in the SCN in one crucial aspect: the SCN neurons in the adult animal express very little GR, if any at all (Ahima and Harlan, 1990; Balsalobre et al., 2000; Cintra et al., 1994; Rosenfeld et al., 1988; van Eekelen et al., 1991a, Van Eekelen et al., 1987a). While the SCN clock is driven by the light-dark cycle, all other cellular clocks in the body and brain depend, besides SCN control, also on CORT acting via GR to regulate PER (Nr1d1, Per1, and Per2) expression, thereby ensuring their circadian alignment. Accordingly, the GR-devoid SCN master clock serves as the pacemaker clock, enslaving the GR-driven cellular clocks elsewhere in the brain and body. See the following references for mechanistic detail (Balsalobre et al., 2000; Lehmann et al., 2023; Oster, 2020; Oster et al., 2016).

Recent research, using more advanced technology, confirmed the absence of GR expression in young adult mouse SCN neurons. However, the GR transcript and protein were progressively more abundantly expressed during development in astrocytes associated with the maturation of the SCN. This is an important observation, particularly because CORT can modulate the SCN clock *in vitro* and *in vivo* via glial gap junctions (Astiz et al., 2022; Cintra et al., 1994; Händler et al., 2024). GR expression in the human SCN has not been demonstrated.

### 2.2 Synchronization of the SCN pacemaker with cellular clocks

The clock mechanism coordinates the daily activities, energy metabolism, and sleep-related events. This includes the timing of sleep patterns, as well as awakening, appetite, food intake, and energy allocation, among many other circadian processes. If CORT secretion aligns with the circadian pattern driven by the SCN, the synchronization of this master clock and all other enslaved clocks is optimal. Such synchronization reinforces CORT's coordination of cell, tissue, and organ function, which underlies optimal stress coping and adaptation (Mavroudis et al., 2012; Mohawk et al., 2012; Nader et al., 2010; Schibler et al., 2016; Spencer et al., 2018; Stalder et al., 2025).

The light-dark cycle involved in melatonin release of the pineal gland is an obvious physical “Zeitgeber*”* (time cue). Other “Zeitgebers” can be social cues, such as meal times, and additional inputs, such as exercise, temperature changes, emotions, or stress experiences. In the human, misalignment of the SCN with peripheral clocks due to phase shifts in the diurnal cycle is common during shift work and jet lag. An altered CORT rhythm also characterizes chronic stress, as demonstrated in animals subjected to chronic defeat stress (Kong et al., 2022; Ota et al., 2021) or in rat offspring exposed to an adverse early-life experience (Walker et al., 2023). Accordingly, such misalignment between the SCN pacemaker lacking GR and the GR-driven peripheral clocks may increase vulnerability to stress-related neuropsychiatric, metabolic, and cardiovascular diseases (Oster, 2020).

**Fig. 1:**
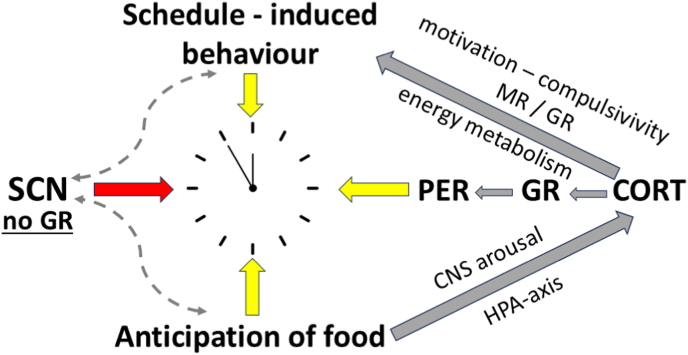
The SCN clock, CORT, and schedule-induced behavior. Schedule-induced behaviors depend on CORT, which may misalign the SCN pacemaker and peripheral clocks via PER induction. Various processes, including e.g., anticipatory feeding, CORT secretion and PER activation, schedule-induced behaviors, motivational arousal, and energy metabolism, activate these peripheral and central clocks. Note that the SCN lacks GR expression (Balsalobre et al., 2000; Rosenfeld et al., 1988; Van Eekelen et al., 1987a). ADX eliminates schedule-induced behaviors, which can be reinstated by either dexamethasone via GR if it concerns wheel-running, and via GR + MR if schedule-induced drinking (Cirulli et al., 1994; Lin et al., 1989). Figure inspired by Henrik Oster, Fig. 4 (Oster, 2020).

An experimental model of misalignment between the SCN pacemaker and the enslaved peripheral clocks, caused by inappropriate CORT action, is the schedule-induced behavior paradigm. In this model, animals on an 80% feeding regimen are fed once per day at a fixed time and readily show phase shifts between the SCN and peripheral clocks. Towards the time of feeding, animals engage in schedule-induced excessive motor (wheel running) activity or excessive drinking (polydipsia) and mount a surge in CORT secretion, which precipitously drops during the consummatory response (Brett and Levine, 1979; Doell et al., 1981; Levine and Levine, 1989). ADX abolishes these schedule-induced behaviors, which are restored by CORT rather than dexamethasone in scheduled-induced polydipsia (Cirulli et al., 1994) and by dexamethasone in wheel running (Lin et al., 1989). This finding suggests an essential role of endogenous CORT in the motivational arousal and compulsivity underlying schedule-induced behavior, which overrides the SCN-driven clock mechanism (Martín-González et al., 2022; Mittleman et al., 1992; Moreno and Flores, 2012) (Fig. 1).

As mentioned, GR expression disappears from SCN neurons around the time of eye opening and innervation by the optic nerve at PND 12, leading to light as a physical “Zeitgeber” rather than circulating CORT (de Kloet et al., 2008; Rosenfeld et al., 1993b). That GR expression is suppressed by establishing innervation has been observed in other systems as well (BOX 2). Before GR disappearance, the pre- and perinatal SCN would respond to maternal circadian CORT variations, either naturally occurring or induced by the steroid given to the dam in drinking water (Catalani et al., 2011; Walker et al., 2023). The findings are supported by recent research showing GR expression in the SCN of 4-day-old pups, which responded to variations in maternal CORT that could be antagonized by mifepristone (Olejníková et al., 2018).

BOX 2 **GR expression depends on neural input to the target cell**

A striking example of nerve input control over GR expression is in the rat pituitary intermediate lobe, which normally lacks GR. Upon pituitary stalk transection, GR becomes expressed in the intermediate lobe, and now CORT stimulates pro-opiomelanocortin (POMC) expression, which fades again when synaptic input is re-established (Antakly et al., 1985; de Kloet et al., 2008; Seger et al., 1988). Other research shows plasticity of GR expression following denervations of the hippocampus and the PVN (De Ronde et al., 1986; Han et al., 2007; Nyakas et al., 1981). The findings support the hypothesis that retinal synaptic input suppresses GR expression in the SCN.

Alt-text: BOX 2

To my knowledge, there are no reports on the developmental pattern of GR expression in the human SCN, although its vasopressin expression appears to respond to CORT treatment (Liu et al., 2006). However, assuming that the rodent findings can be extrapolated to humans, it is of interest to combine real-time measurements of CORT levels in blood with SCN activity. Today, this is feasible through automated ambulatory CORT measurement of samples obtained from interstitial fluid in subcutaneous fat (Klaas et al., 2025) or sweat (Liu et al., 2025). This would allow correlating CORT rhythms with SCN activity, thereby optimizing CORT replacement chronotherapy in adrenally deficient individuals (Russell et al., 2024).

## 3 Maternal care

**Gig discovered that early handling during the first two postnatal weeks protects against the adverse effects of a mild electric shock** (Levine et al., 1956)**. Here, two studies are reported that are inspired by this key finding. Firstly, neonatal handling, which induces maternal care, protects the pup against the potential noxious impact of perinatal dexamethasone treatment** (Claessens et al., 2012b)**. Secondly, the quality of maternal care, as measured by licking and grooming, is an essential determinant of later-life cognitive, emotional, and neuroendocrine responses to an acute stressor, a phenomenon known as the predictive adaptive response, also referred to as the match/mismatch hypothesis** (Champagne et al., 2008, 2009; Claessens et al., 2011)**.**

The handling of pups triggers nursing behavior in the dam upon reunion, including arched back nursing posture, licking, and grooming (Weinberg and Levine, 1977). The higher the licking and grooming (LG) score, the more beneficial the behavioral and neurochemical outcomes (Meaney, 2001). Early handling effects are non-genomic (Denenberg and Whimbey, 1963) and their epigenetic signature can be quantified as the degree of methylation, e.g., of the GR, and histone modification for enduring changes in chromatin organization, also in the human brain (McGowan et al., 2009; Meaney et al., 2007; Weaver et al., 2004). The findings substantiate the maternal mediation hypothesis in shaping the offspring's later-life phenotype, where it takes two to tango: maternal behavior and the pup's coping ability (Macrì and Würbel, 2006).

In a series of groundbreaking studies, Regina Sullivan's research showed that CORT acts as a developmental switch in the amygdala during attachment learning. Pups learn in the first week of life to secure attachment to the mother via a pleasant odor, even when this is linked to an aversive event (i.e., an electric shock), but this does not occur in the second week when the electric shock evokes fear. The substrate of odor/shock attachment learning is a locus coeruleus–olfactory bulb circuit, which is altered by a CORT-driven switch enhancing amygdala activity underlying fear. CORT can just be injected into the circulation or locally in the amygdala, or elevated by procedures such as the limited bedding and nesting paradigm. At the same time, the switch is blocked in the amygdala by the GR antagonist mifepristone. Subsequent studies showed that odor/shock attachment learning reduces later-life fear learning, which is in line with the “predictive adaptive response” hypothesis (section 3.2.) (Moriceau et al., 2004, 2006, 2009, 2010; Moriceau and Sullivan, 2006; Rice et al., 2008).

### 3.1 Early handling protects against the perinatal dexamethasone outcome

We learned the power of the maternal mediation hypothesis in early-life dexamethasone experiments. It is clinical practice to apply either antenatal or directly after birth dexamethasone to prematurely born children to promote lung maturation. While essential for survival, the same treatment is known to have severe side effects. Animal experiments were particularly ominous, predicting even a 25% reduction in lifespan, while causing hypertension and cognitive decline, and compromising immune function and metabolism in the dexamethasone-treated offspring (Kamphuis et al., 2007; van de Loo et al., 2024).

In our rodent model, dexamethasone was administered subcutaneously (sc) to rats at a tapering dose during the first 3 postnatal days to mimic a clinical treatment regimen for prematurely born infants, corresponding to the third-trimester developmental stage, weeks 26-32 of gestation. In these first experiments, we observed significant reductions in body weight at PND 10, accelerated eye opening from PND 12 to PND 8, decreased hippocampal cell proliferation, and reduced glial cell number. Intracerebroventricular administration of the GR antagonist mifepristone to the pups prevented the dexamethasone effects in the developing brain (Claessens et al., 2012a).

However, in a longitudinal study design examining young adult, middle-aged, and senescent animals, there was no effect of the early-life dexamethasone treatment on the endocrine and behavioral phenotype. Additionally, the previously reported 25% reduction in survival, from 29 to 21 months, was not observed. For a PhD student, a nightmare. But! We realized that the difference from other studies might be due to the experimental design. All other published studies had used a ‘whole’ litter design for the treatment groups, and the pups were barely touched in the animal house. We applied a ‘split litter’ design, allowing better comparison of the different treatment groups within individual litters. Thus, our procedure involved daily weighing and marking each individual = handling (Claessens et al., 2012b).

In a subsequent study, the effect of dexamethasone was tested in handled and non-handled animals. In all groups, dexamethasone advanced early eye opening and reduced pup body weight. In adulthood, non-handled animals were more susceptible to the adverse effects of early dexamethasone exposure than handled animals, as indicated by spatial learning. In stress-induced CORT response, acoustic startle reactivity, pre-pulse inhibition, and fear acquisition, the handled animals performed best, and the effects were attenuated by prior dexamethasone, whereas the non-handled groups performed worst. The effect demonstrates that the outcome of neonatal dexamethasone treatment is not deterministic but strongly interacts with components of the postnatal environment (Claessens et al., 2012b; de Kloet et al., 2014).

### 3.2 The predictive adaptive response

The positive long-term handling effect actually occurs due to increased LG by the mother when the newborn is returned to the cage (Liu et al., 1997). Moreover, low LG impaired spatial learning and object recognition in the offspring. Still, this effect could be reversed by cross-fostering with dams displaying high LG, strongly suggesting a key role for maternal care, i.e., the maternal mediation hypothesis (Champagne et al., 2003; Francis and Meaney, 1999; Liu et al., 2000), but see the finding of Rixt van der Veen showing that cross-fostering with high-LG dams does not always override genetic predisposition for addictive behavior, stress coping, and hippocampal neurogenesis (Koehl et al., 2012; Van Der Veen et al., 2008).

If the biology of the offspring of high-LG is compared to that of low-LG dams, a few very interesting notions were made by Danielle Champagne. First, the young adult high-LG offspring not only secreted less CORT with faster shut-off in response to a novelty stressor, but also showed increased hippocampal MR and GR expression, as well as increased hippocampal dendritic arborization and spine number (Champagne et al., 2008). Secondly, long-term potentiation in hippocampal CA1 neurons was markedly enhanced in well-groomed offspring compared with neglected pups, in agreement with previous findings (Bredy et al., 2003).

**Fig. 2:**
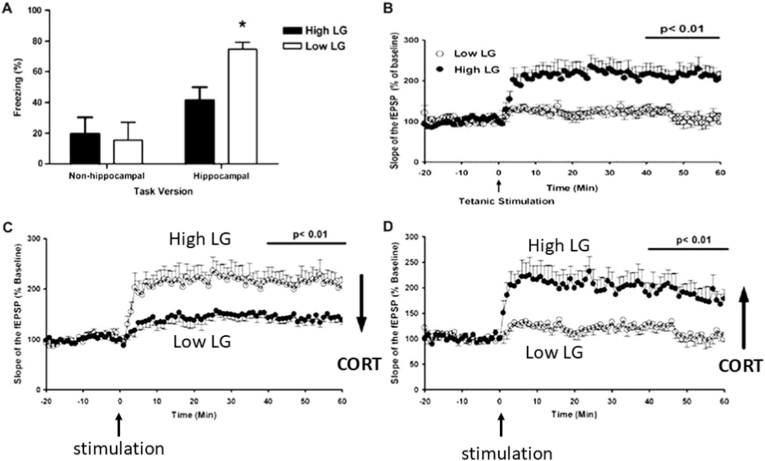
CORT exposure changes hippocampal long-term potentiation (LTP) of low- and high licking and grooming offspring in opposite directions. Young male adult high-LG offspring secreted less stress-induced CORT, showed a higher number of hippocampal MR and GR, and increased dendritic complexity in comparison with low-LG rats. They also differ profoundly in hippocampal LTP *in vitro*, which predicts their fear-conditioning behavior (Champagne et al., 2008). (A) When contextual fear learning was assessed 24 h after conditioning, Low-LG animals showed significantly greater freezing in the conditioned context than high-LG animals in the hippocampal-dependent version of the task. i.e., the situation where animals were allowed to form a spatial representation of the shock environment.∗P < 0.05 (Student's t-test). (B) Synaptic potentiation is impaired in low versus high-LG offspring under basal conditions. Tetanic stimulation (10 Hz, 900 pulses) of Schaffer collateral afferents significantly potentiated the slope of the field excitatory postsynaptic potential (fEPSP) in high but not low-LG offspring. The bar represents the last 20 min of recording. (C) Compared with vehicle conditions, a significant reduction in the percentage of fEPSP was prominent in slices from high-LG offspring pre-treated with 100 nmol/L of corticosterone. (D) In contrast, slices from low-LG offspring did not display reduced but enhanced long-term potentiation in response to a high dose of corticosteroid. The bar represents the last 20 min of recording (reprinted from (Champagne et al., 2009, 2008).

A new finding was that when the hippocampal slice from the high-LG animals was exposed to corticosterone, LTP dramatically deteriorated. In contrast, LTP from the low-LG animals improved towards that of the untreated high-LG offspring (Fig. 2). Finally, the well-groomed animals performed poorly in hippocampal-dependent contextual aspects of fear-motivated behavior, in contrast to the excellent performance of the low-LG littermates (Bagot et al., 2009; Champagne et al., 2008).

Further support came from studies showing that, in addition to the hippocampal CA1, the dentate gyrus also showed reduced dendritic complexity in low-LG offspring. LTP was lower, but rapidly increased *in vitro* with CORT and the β-adrenergic agonist isoproterenol, an action that involved increased NMDA receptor activity. Hippocampus-related contextual, rather than amygdala-cued, fear conditioning was enhanced in low-LG offspring, while hippocampus-associated spatial learning was impaired (Bagot et al., 2009, 2012; Liu et al., 2000).

In a further refinement of experimental design, Sara Santarelli and colleagues compared in male Balb/c mice the outcome of either the ‘early handling’ supportive procedure with the chaotic, adverse early life experience in the limited bedding and nesting paradigm (Birnie and Baram, 2025; Gilles et al., 1996; Korosi and Baram, 2010; Rice et al., 2008; Walker et al., 2017). At adulthood, the animals were either housed for three weeks in a supportive environment or exposed daily to social defeat. The outcome of this elegant experiment supported the predictive adaptive response hypothesis: brain GR expression increased, HPA axis activity decreased, and fear-motivated behavior was affected in both male and female matched conditions (Nederhof and Schmidt, 2012; Santarelli et al., 2014, 2017).

Nikos Daskalakis and coworkers found support for the mismatch hypothesis by including groups that, after classification of the rats into high- or low-LG, were either kept post-weaning in groups or in isolation as an additional ‘hit’. The high-LG combined with social isolation showed enhanced responsivity, translated to “psychosis susceptibility”, if tested for apomorphine responsivity and pre-pulse inhibition (Daskalakis et al., 2012).

The above findings on behavioral programming support the predictive adaptive response hypothesis, which is based on another (feeding) aspect of maternal behavior. It was demonstrated that perinatal nutrient availability provided by the dam prepares for the later-life metabolic performance of the offspring (Gluckman et al., 2008; Hanson and Gluckman, 2014) (BOX 3).

BOX 3 **Developmental hypothesis for programming brain and body functions**

(i) David Barker noted a correlation between low birth weight and the development of obesity and hypertension in later life. In subsequent studies, it was found that a poor nutritional state in the first three months of pregnancy had long-term adverse effects, particularly when nutrition was abundant later in life. This key finding led to the Developmental Origin of Human Adult Disease (DOHAD) (Barker, 1990).
(ii) Peter Gluckman and Mark Hanson developed the predictive adaptive response (PAR) hypothesis. It proposes that “a fetus "forecasts" its future environment based on signals from the mother, such as nutrient availability, and adapts its development accordingly to suit those predicted conditions better” (Gluckman et al., 2008; Hanson and Gluckman, 2014).
(iii) Danielle Champagne and associates formulated the match/mismatch or predictive adaptive response hypothesis as a new perspective in the field of psychiatry: “early-life conditions may prepare for life ahead through glucocorticoid programming and phenotypic plasticity to ‘match’ future environmental demands. This concept has led to the hypothesis that a ‘mismatch’ between early and later life experiences can enhance vulnerability to disease (Champagne et al., 2008, 2009; Daskalakis et al., 2013; Oitzl et al., 2010). Or as stated by Mathias Schmidt: “Overall, our data support the notion that being raised in a stressful environment prepares the offspring to better cope with a challenging adult environment.” (Nederhof and Schmidt, 2012; Santarelli et al., 2014, 2017). How such early life programming of stress coping drives individual differences in cognitive aging is an open question (de Kloet and Oitzl, 2003; Oitzl et al., 2000)
(iv) Boyce and Ellis distinguished two subtypes in stress coping based on genetic background, defining the susceptibility to environmental challenges in relation to the nature and timing of experiences during development. They distinguished individuals who are either more susceptible or resistant to the environmental input using the orchid and dandelion metaphors, respectively. The concept predicts a U-shaped curve, consistent with either the cumulative stress or the predictive adaptive response hypotheses, rather than the unitary stress diathesis theory (Ellis and Boyce, 2008).

Alt-text: BOX 3

## 4 Maternal deprivation

**Gig observed as early as 1957 that “the thermo-tactile contact between mother and infant is yet another mechanism that further inhibits the infant's capacity to elevate CORT ”** (Levine, 1957; Stanton et al., 1988). **This section elaborates on this concept using either a single 24-h maternal deprivation or three consecutive daily separations of 8 h each. This ‘maternal deprivation’ approach could distinguish the infant's central components of the stress response system engaged with sensory (thermo-tactile) contact from CORT secretion driven by metabolic need. It was shown that the pup can predict the dam's return the next day after a single 8-h experience of isolation, using CORT secretion as the criterion (see 4.3). We conclude that maternal deprivation primes the amygdala to generate an adult fearful phenotype and profoundly enhances individual variability in cognitive performance over the life span.**

### 4.1 GR-mediated pituitary feedback in the stress hyporesponsive period

There is a stress hyporesponsive period (SHRP) in the early life of rats (PND 4-14) and mice (PND 1-10) during which relatively mild stressors do not trigger a CORT response, as they do in adult animals (Levine, 2005). Low free CORT levels with small fluctuations characterize the SHRP because circulating CBG is absent in the first two weeks of life. CBG is high around birth, then decreases to near-detectable levels for 2 weeks, after which its liver expression and blood concentration rapidly increases again (Elfahime et al., 1992; Laryea et al., 2013; Moisan and Castanon, 2016; Toews et al., 2021).

Hypo-responsiveness of the adrenal to stress-induced ACTH is the most proximal cause of the SHRP. The underlying reason for this stress hypo-responsiveness is the CORT feedback action on the HPA axis, notably the pituitary corticotrophs (Walker et al., 1986). Matthias Schmidt demonstrated during his PhD in Leiden that pituitary CORT feedback was indeed crucial for maintaining the low CORT levels by showing disinhibition of the HPA axis after mifepristone administration to the pup (Schmidt et al., 2005). In subsequent studies, using a mutant with a selective anterior pituitary GR deletion in corticotrophs, CORT levels during the SHRP are also elevated. Surprisingly, in adulthood, the HPA axis activity of the deletion mutant is not significantly different from that of the controls. Still, the mutants show an altered behavioral response to an inescapable stressor, such as during forced swim exposure (Schmidt et al., 2009).

A reminder in these studies is that GR-mediated feedback can only be demonstrated if CORT secretion increases during stress, even though this increase may be very small during the SHRP. If mifepristone was offered ‘stress-free’ in oatmeal, basal CORT secretion – as can be predicted – does not increase in the newborn pup. Likely, under basal conditions, MR maintains circulating CORT in a *proactive* control rather than the GR-mediated *reactive* mode. ‘Proactive’ implies that the HPA axis is prevented from being activated, while ‘reactive’ refers to the negative feedback action of CORT to terminate the stress response (Enthoven et al., 2008; Ratka et al., 1989; van Oers et al., 1998).

### 4.2 Dissociation of central and adrenal stress responses during development

Upon removal of the dam for 24 h at PND 3-4, CORT levels gradually increase over the 24-h period, initially in conjunction with ACTH until 8-12 h, after which ACTH declines towards pre-separation levels (Schmidt et al., 2005). The response to a novelty stressor also gradually increases in magnitude over the 24-h deprivation period (Stanton et al., 1988). Based on this 24-h separation model, several discoveries were made with Gig, each deepening insight into the impact of a pup's day without care.

*Firstly, the opposite outcome of early vs. late 24-h maternal deprivation at weaning is related to sensory input rather than CORT*. Helga van Oers, who conducted her PhD research at Stanford and Leiden Universities, showed that the outcome of maternal deprivation on PND 3-4 was the opposite of that observed on PND 11-12 when measured at PND 20. Early deprivation at PND 3-4 led to increased activation of stress-induced pituitary ACTH release, PVN-CRH- and c-fos mRNA expression at PND 20. In contrast, the same markers were lower than control values in 20-day-olds deprived at PND 11-12.

Thus, the 24-h deprivation experience during SHRP gives an opposite outcome between the first and second postnatal week when measured at weaning. These long-term effects were due to a different impact of the lack of nurturing behavior, as the suppression of deprivation-induced CORT secretion with dexamethasone at either time, PND 4 or PND 12, did not affect the long-term outcome at PND 20 (Van Oers et al., 1998a, 1999). The different outcomes of manipulations during the first or second postnatal week represent different rates of maturation, which has been further elaborated as a model for anxiety disorders (Bath et al., 2016; Peña et al., 2019).

**Fig. 3:**
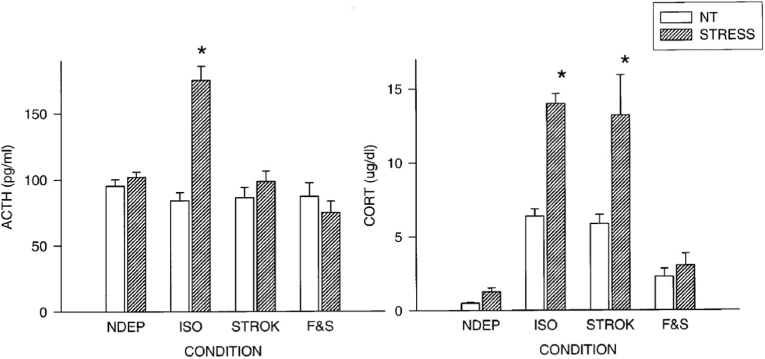
Immediate effects on ACTH and CORT of feeding and stroking pups deprived for 24 h from maternal care. Plasma ACTH (picograms per milliliter) and CORT (micrograms per deciliter) levels in 12-d-old pups, both under basal conditions (open bars, NT = no treatment) and 30 min after a saline injection (closed bars, STRESS). Litters were deprived for 24 h on PND-11, during which time they were either left undisturbed (ISO), stroked (STROK), or stroked and episodically fed (F & S) (*n=*10–12 per group). NDEP animals served as controls. ∗*p* < 0.05, significant from NDEP counterparts. From Helga van Oers, PhD Thesis (17-06-1998): *Maternal deprivation: implications for the ontogeny of the neural stress circuitry* (van Oers et al., 1998b).

Secondly, *sensory input regulates central components of the stress response system, and energy demand directly controls CORT secretion*. If, in the maternally deprived rat, the licking and grooming was mimicked with brief (45 s) anogenital stroking using a warm wet painter's brush at 8 h following the initiation of the deprivation procedure, and repeated twice at 16 and 24 h, the *central* component (PVN CRH- and c-fos mRNA and hippocampal MR mRNA) of the stress-induced HPA axis, including ACTH release, was restored towards control levels, while the CORT response to the 30-min novelty exposure remained. If the pup was also given milk 3 times every 8 h during the 24-h deprivation period, the increased stress-induced CORT levels and the decreased brain GR mRNA expression were normalized. These changes in CORT and GR were dependent on nurturing behavior, since suppression of the stress/deprivation-induced pituitary ACTH and adrenal CORT secretion by dexamethasone did not affect CRH and c-fos mRNA responses in the hypothalamus. The complementary effects of feeding and stroking were not only observed during the 24-h deprivation period but also persisted until PND 20 (Rosenfeld et al., 1993a; Suchecki et al., 1993; Van Oers et al., 1999, 1998, 1998b) (Fig. 3).

This striking finding clearly shows that during the SHRP, a central sensory component responds to stressors, as supported by other studies using a similar design with hypothalamic c-fos, CRH, and vasopressin mRNA expression as endpoints (Dent et al., 2000; Vazquez et al., 2006). These central responses then likely result in epigenetic regulation of, for instance, the GR (Meaney et al., 2007). In addition, a peripheral energy-metabolism component regulates CORT secretion. Indeed, during the CORT rise induced by deprivation, plasma glucose and leptin levels decreased, while circulating ghrelin and arcuate NPY mRNA levels increased. The increased CORT secretion could be suppressed again in the 24h-maternally deprived animal by normalization of glucose levels or by administration of a ghrelin antagonist, but not by leptin (Schmidt et al., 2006).

The finding aligns with the important observation by Mary Dallman and coworkers that in adult animals, glucose can act as a substitute for CORT to maintain HPA-axis activity (Akana et al., 1994; Dallman et al., 1994). These hallmark contributions by Mary Dallman and coworkers are fundamental for understanding the role of energy metabolism in the so-called *glucocorticoid feedback paradox:* glucocorticoid administration suppresses a subsequent stress-induced rise in CORT, while a stress-induced CORT rise to a similar level does not (Dallman, 2010, 2011; Dallman and Jones, 1973; de Kloet, 2023).

### 4.3 Prediction of maternal care matters for the pup's control of CORT secretion

The following experiment, performed by Leo Enthoven, yielded a surprising outcome that, at the same time, provides a valuable insight into the pup's stress response system. Firstly, if dam removal was limited to 8 h a day starting at PND 3, there was an apparent rise in CORT after the first separation, which disappeared after the same separations on PND 4 and 5. Plasma ghrelin levels increased, and glucose levels decreased after each daily separation, which excludes a role of energy metabolism. The prediction of maternal return is so strong that it also holds for rodents isolated for 8 h each day in a novel environment (Daskalakis et al., 2011; Enthoven et al., 2008).

That prediction of maternal return after one separation experience prevents HPA axis activation the next day is also demonstrated by administration of a GR antagonist, which triggers a profound ACTH and CORT response after the first 8-h separation on PND 3, but not so at PND 4 and 5, because there is no stress-induced CORT feedback to be antagonized. An MR antagonist still caused a slight increase in CORT and ACTH after the 3rd separation, in line with the previously mentioned MR-mediated *proactive* control. Accordingly, we believe this action mediated via the MR is because the pup, after experiencing the first separation (PND 3), can already *predict* the mother's return the following days (PND 4 and 5) after a similar period of her 8-h absence, even in the face of increasing metabolic demands (Enthoven et al., 2008).

Secondly, after each daily 8-h separation, CORT, ACTH, and PVN c-fos mRNA still responded to a 30-min novelty stressor. Thus, the newborn's HPA axis readily desensitizes to repeated 8-hr daily maternal separation, but continues to respond to a novelty stressor presumably via a central MR-mediated mechanism rather than a GR-mediated one.

Nikos Daskalakis used the repeated 8-h separation model from PND 3, 4, and 5 to compare outcomes in animals isolated in the home environment as a group or individually housed in a novel cage. He reproduced the Enthoven data on the power of the pup's prediction. He also found that pups experiencing novelty alone still showed increased c-fos mRNA expression in the amygdala after the last separation, which persisted into adulthood. Amygdala priming appeared to be associated with increased emotional reactivity and better memory performance. At the same time, social interaction decreased along with increased stereotypy and impaired sensorimotor gating (Daskalakis et al., 2012, 2013, 2014).

### 4.4 Early life manipulations: effect on aging

Early handling of Long-Evans rats affects their aging. Michael Meaney et al. (1988) demonstrated that, as a group, these handled animals secreted lower levels of CORT and improved cognitive performance at senescence. Previously, Phil Landfield found that adrenalectomized (ADX) animals replaced with low levels of CORT, just sufficient to maintain MR occupancy, were protected against age-related declines in various hippocampal and cognitive features of aging (Landfield et al., 1978). Jonathan Seckl and Joyce Yau discovered that deleting the 11β-Hydroxysteroid dehydrogenase (HSD-1) gene in the brain (BOX 4) to reduce intracellular CORT attenuated age-related cognitive decline (Yau and Seckl, 2012). Collectively, these studies suggest that ‘healthy’ aging is associated with low circulating CORT levels, while age-related diseases may develop because of a hippocampal GR-dependent deficit in turning-off the stress response (Sapolsky et al., 1986).

BOX 4 **11-oxidoreductases are an intracellular determinant of bioactive CORT**

11β-Hydroxysteroid dehydrogenase type 2 (HSD-2), which degrades CORT, was identified as an intracellular enzymatic mechanism that determines the MR's aldosterone specificity in epithelial cells that retain Na+, such as in the kidney and in the n. tractus solitarii in the brain stem (Edwards et al., 1988; Evans et al., 2016; Funder et al., 1988; Gasparini et al., 2018; Geerling et al., 2006). Alternatively, HSD-1 is a reductase that regenerates CORT intracellularly and underlies CORT's preference for the MR in non-epithelial cells, e.g., brain, heart, and skin (Chapman et al., 2013).

Alt-text: BOX 4

Meanwhile, an age-dependent decline in hippocampal MR expression appeared also prominent at senescence, which could be counteracted by treatment with a neurotrophic ACTH 4-9 analog ORG 2766, as well as, surprisingly, the bio-active ingredient of Ginseng, the Ginsenoside RG1 (De Kloet et al., 1987; Reul et al., 1988; Rigter et al., 1984). Studies in the dog also revealed an age-induced decrease in brain MR capacity, and increased basal and stress-induced HPA axis activity (Rothuizen et al., 1993). In humans, stimulation of the MR in older adults was found to enhance cognitive performance (Hinkelmann et al., 2015; Otte et al., 2015). Brain MR activation is known to affect cognitive flexibility, the selection of coping style, and the control of the onset rather than the termination of the HPA axis (de Kloet et al., 2016; Joels et al., 2012; Schwabe et al., 2022).

In a series of experiments with Judith Workel and Melly Oitzl, we used Brown Norway rats because these animals remain healthy for 33-36 months until passing away. As expected, the Brown Norway's have low circulating CORT levels during senescence (van Eekelen et al., 1991a, van Eekelen et al., 1991b; Van Eekelen et al., 1995; Workel et al., 2001). Brown Norway MR harbors a mutation that has made it more responsive not only to CORT but also to progesterone (Marissal-Arvy et al., 2004). This MR gain-of-function results in enhanced *proactive* control of the HPA axis, which may explain their low circulating CORT levels.

In our aging study, 80 litters with a number of >4 and <7 male pups were used. Half of the pups in each nest were 24 h maternally deprived from PND 3-4, while the other half served as controls that stayed with the dam. CORT, ACTH, CRH, MR, GR, BDNF, and various behaviors, including cognitive performance (learning the Morris water maze), were measured at 3 months, 12 months, 24 months, and 30-32 months of age in a longitudinal and cross-sectional (transversal) design.

**Fig. 4:**
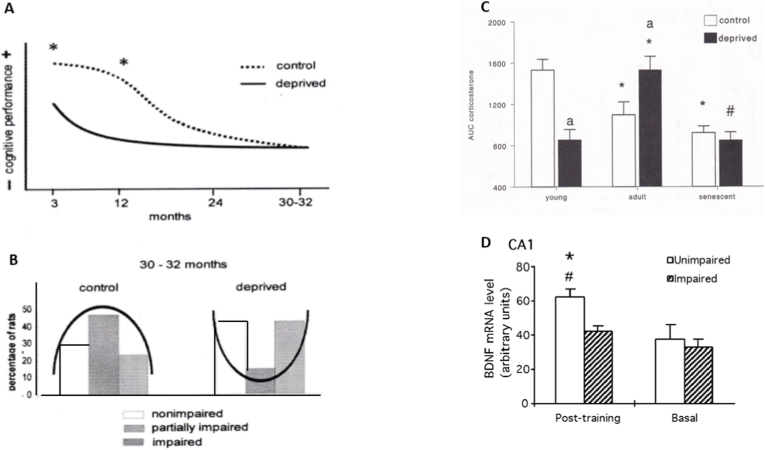
From birth to senescence: cognitive performance, CORT plasma levels, and hippocampal BDNF. Male Brown Norway rats were maternally deprived at PND day 3 for 24 h (deprived) or were maternally reared (control). 80 litters with >4 and < 7 male pups were used in transversal and longitudinal study designs and examined at 3, 12, 24, and 30-32 months. At senescence, 63 non-deprived and 73 deprived animals survived (80-83% survival). 102 animals participated in the behavioral experiments, which were also used to analyze brain MR, GR, CRH, and BDNF expression after the behavioral analyses. A: Note the impairment of performance of the maternally deprived animals in the spatial learning test at 3 and 12 months of age (Morris water maze). At senescence, control (n = 23) and deprived rats (n = 26) are partially impaired and, on average, not different (Oitzl et al., 2000). B: The maternally deprived rats display, during senescence, enhanced individual variation in performance. The majority of the controls are partially impaired in learning the Morris water maze. In deprived animals, aging drives cognitive performance to extremes: deprived senescent rats are either predominantly impaired or not, at the expense of a partially impaired average. The finding was replicated with separate groups of controls (n = 11) and deprived animals (n = 21). On the y-axis, the percentage of animals is given that are either non-impaired, partially impaired, or impaired (de Kloet and Oitzl, 2003; Oitzl et al., 2000). C: Total amount of CORT (μg/dl) secreted in blood during 120 min exposure to a novel environment in young, adult, and senescent control (open) and deprived (black bars) rats. Areas under the curve are adjusted for basal levels. The increased CORT output is due to a prolonged secretion, suggesting feedback resistance. Data represent the mean ± SEM (P < 0.05); # versus adult deprived rats; a) vs controls of the same age (Workel et al., 2001). D: At 3 h after learning the Morris water maze, BDNF mRNA levels were increased in the CA1 of non-impaired performers in comparison to levels of the impaired group. ∗Significantly different from basal level (*P*#0.05) (Schaaf et al., 2001). This work was presented in the PhD theses of Judith O. Workel (1 999), Maternal Deprivation: Implications for Stress, Cognition, and Aging, and Marcel PM Schaaf (1999), Corticosterone, BDNF, and memory formation (both at the University of Leiden, The Netherlands). The figure is a composite of this PhD work as presented in (de Kloet and Oitzl, 2003) and cited in the legends of panels A, B, C, and D.

The deprived male animals were impaired in learning at 3 and 12 months of age. At senescence, control and deprived animals showed, on average, partial impairment of spatial learning in the Morris water maze. However, upon inspection of individual performance, the deprived littermates showed a bimodal distribution: about half performed excellently, while the other half deteriorated, and only a few showed a partial decline; such partial decline was typical for most controls. In a replicate sample, this finding was reproduced: the deprived animals were predominantly good or poor at the spatial cognitive task, at the expense of median performance, as commonly observed in the partially impaired controls. Apparently, adverse early-life experiences amplified individual differences in cognitive performance at senescence. Interestingly, better performance correlated with increased hippocampal BDNF expression (de Kloet and Oitzl, 2003; Oitzl et al., 2000; Schaaf et al., 2001; Workel et al., 1997, 2001) (Fig. 4).

A systematic review of the literature supports the finding of enhanced age-related individual differences in cognitive performance (Bonapersona, 2022). At the time, in the late eighties, I remember the discussions we had with Gig about the notion that, apparently, early handling protected all pups from cognitive decline. The data presented by Melly Oitzl (Oitzl et al., 2000) suggest that the 24-h deprivation procedure enhances individual variation within the deprived group rather than affecting all animals. How is that possible?

A clue towards answering this intriguing question came from another interesting phenomenon. At midlife, all animals of the deprived offspring showed an increased and prolonged stress-induced CORT secretion. Then, stress-induced CORT levels declined and were indistinguishable from those of controls at senescence; their individual CORT levels were not correlated with cognitive performance. We suggested that this period of increased midlife stress activates a so far unknown mechanism that drives cognitive aging toward either excellent cognitive aging or senility (de Kloet and Oitzl, 2003; Oitzl et al., 2000).

Carmen Sandi tested the hypothesis that *mid-life stress* affects cognitive aging. For this purpose, male Wistar rats were first divided at 4 months of age into low- or high-reactivity groups based on their responses to novelty, and then, at midlife (12 months), exposed to 4 weeks of chronic stress or left undisturbed. Interestingly, at 18 months, the high-reactivity group showed *impaired* performance in the spatial learning task compared with their unstressed controls and the low-reactivity stress rats. This finding supports the notion that a high stress reactivity group at midlife shows increased vulnerability to cognitive decline (Sandi and Touyarot, 2006). It did not, however, demonstrate that early adversity combined with midlife stress may enhance pre-existing phenotypic differences in resilient and vulnerable individuals.

### 4.5 Prenatal research and sex differences

To my knowledge, Gig did not engage very much in prenatal research; there were too many variables, and it was too complicated, he commented when asked. Research on the effects of prenatal stress is, of course, very important, as is clearly demonstrated by the Stefania Maccari lab. The model includes daily repeated exposure of pregnant rats from day 11 of gestation to restraint stress and bright light until delivery. The stressor is of perinatal nature, since the stressed pregnant dams also display reduced maternal care after delivery. It is a model with face validity for anxiety and demonstrates sex differences in circadian organization of behavior, as well as in stress-induced changes in stress coping and cognitive function (Koehl et al., 1997; Morley-Fletcher et al., 2019; Vallée et al., 1997).

Prenatal influences may start, however, as early as the implantation of the blastocyst in the uterine lining. “*Actually, large-scale epidemiological studies assessing the impact of artificial reproduction technology (ART) on offspring health at birth and in adulthood are urgently required”* (Sciorio et al., 2025). The authors are referring to the embryo's incubation conditions and the factors that determine implantation in the uterus. (De Kloet et al., 2005a; Helmerhorst et al., 2007; Sibug et al., 2005). Alternatively, studies by the Isabelle Mansuy and Johannes Bohacek labs at the ETH in Zürich, Switzerland, show that chronic stress can even be passed on to subsequent generations and that this transgenerational programming likely proceeds via an epigenetic mechanism (Bohacek and Mansuy, 2015).

Sex differences in early-life programming were also not the primary focus of Gig's research, even though we observed profoundly divergent sex-dependent development of hippocampal CORT receptors (Sutanto et al., 1996). Hippocampal structure and neurogenesis showed a sex difference after 24 h of maternal deprivation on PND 3 (Oomen et al., 2009). Today, research focuses on sex-dependent developmental changes in limbic cortical circuitry using the limited bedding and nesting model, for instance, on CRH neurons in the central amygdala or the hippocampus (Bath, 2020; Jiang et al., 2025; Sanguino-Gómez and Krugers, 2024). This model reveals enhanced fear responses in adult females rather than in males, associated with increased metabolism in the basolateral amygdala and hippocampus, including a role for the FKBP5-GR/MR complex (Bordes et al., 2024; van Doeselaar et al., 2023). Studies on sex differences in the outcome of early life adversity are very much needed, but indeed complex, particularly because of the profound sex difference in coping strategy from fetus to adulthood, culminating in preferred female tend-and-befriend social coping vs the rather male fight-or-flight responses to stress (Bangasser and Cuarenta, 2021; Bangasser and Valentino, 2014; Hodes et al., 2023; Luine and Frankfurt, 2020; Sandman et al., 2013; Taylor et al., 2000; ter Horst et al., 2013).

## 5 Concluding remarks

This contribution to the special issue commemorating Gig's 1925 birth year highlights our 20 years of collaborative research. We agreed during a conference in Norway back in 1986 that we would exploit the new knowledge on CORT receptorology to learn more about how the infant copes with life. During these two decades, I witnessed and enjoyed familiar traits of Gig, but above all, it was always the experimental design that interested him most. It was a great joy to visit him and his colleagues at Stanford, in the wooden emergency building, to become embedded in the science of the developing brain.

This article summarizes some highlights of PhD and postdoctoral research in which Gig served as an advisor, which include:

(i) The disappearance of SCN neuronal GR-expression at early life may underlie later life circadian phase dissociation. Balsalobre's publication litterally served as an "eye-opener" in this respect (Balsalobre et al., 2000; De Kloet et al., 1988; Rosenfeld et al., 1988, 1992, 1993b; van Eekelen et al., 1991a; Van Eekelen et al., 1987; Lehmann et al., 2023).
(ii) The recognition that the SHRP's most proximal cause is the atrophied adrenal, which can be reactivated, however, during metabolic emergency. That the brain remains responsive during the SHRP to sensory stimulation all the time was an “aha experience.” (Ferreira de Sá et al., 2023; Suchecki et al., 1993; van Oers et al., 1998). This finding led to the awareness that a few-day-old pup can already predict, after a single 8-h experience of isolation, the dam's return the next day. This ability to predict safety and food is sufficient to keep its stress system at bay (Daskalakis et al., 2011; Enthoven et al., 2008).
(iii) Then, the discovery of the opposite hippocampal LTP response to CORT exposure observed in high-LG vs. Low-LG offspring as an inroad to the predictive adaptive, match/mismatch, or double hit hypothesis (Champagne et al., 2008; Daskalakis et al., 2013; Nederhof and Schmidt, 2012; Santarelli et al., 2014, 2017; Bagot et al., 2009).
(iv) Finally, the profound individual variation in cognitive performance of maternally deprived senescent Brown Norway rats, which might have been driven by a midlife crisis in animals condemned to living for almost three years in a cage (Oitzl et al., 2000). These were all fascinating discoveries thanks to Gig's original take on data.

The knowledge gained from Gig has been helpful in my support of Megan Galbally and coworkers at Monash University, Australia, on the role of CORT in the programming of childhood internalizing and anxiety disorders until today. The data are obtained from the Mercy Pregnancy and Emotional Wellbeing Study (MPEWS), which is a longitudinal cohort study exploring the impact of depression experienced from conception until birth on 887 women and their children. This multi-disciplinary endeavour uncovered that maternal depression is associated with increased CORT reactivity in the offspring at 12 months of age, which was found to predict internalizing problems and anxiety at 4 years of age (Galbally et al., 2021a, 2021b, 2022a, 2022b, 2023). This finding is in line with previously reported CORT-driven changes in growth and connectivity of the right amygdala in female offspring of depressed mothers (Buss et al., 2012; Graham et al., 2019; Soe et al., 2018). The underlying cause of this female-specific amygdala programming may well be the increased fetal bioavailability of CORT resulting from suppressed placental 11-HSD-2 activity and epigenetic modification of the receptors that mediate CORT action.

‘*I have never been able to eliminate the error bars*,’ Gig stated in his marvelous 2005 overview (Levine, 2005), in the awareness of the significant variation in the outcomes of early-life studies. Such variation becomes even more impressive if individual pups, rather than groups, are analysed in response to maternal care (Claessens et al., 2011; van Hasselt et al., 2012). Moreover, there is an urgent need to exploit previously published data. Using a 3-level mixed-effects meta-analysis and MetaForest, a novel machine-learning approach for thoroughly exploring heterogeneity, data from more than 400 independent experiments are readily accessible via MaBapp (https://osf.io/ra947/), allowing researchers to run tailor-made meta-analyses. This meta-analysis demonstrated that early-life adversity increases individual variation in stress coping and adaptation: such adversity increases anxiety-like behavior and decreases social behavior, while promoting memory formation in later life under stressful conditions, and impairing non-stressful cognitive performance (Bonapersona et al., 2019).

What will the future bring? One fascinating challenge arises with the advance of deep learning, which enables pose estimation of behavior from video recordings of behaving rodents (DeepLabCut). This enables unprecedented (machine-learning-driven) behavioral phenotyping analysis of stress-coping styles with the aim of revealing hitherto unknown behavioral patterns (Kovarova et al., 2025; Sanguino Gómez, 2025). No doubt that the application of this new technology will illustrate the lesson learned from Gig: “*nothing is written in stone*”.

## Declaration of competing interest

ER de Kloet has nothing to declare.

## Data Availability

No data was used for the research described in the article.
